# Multistage assessment of construction delay factors using expert evaluation and real project data

**DOI:** 10.1038/s41598-026-53262-4

**Published:** 2026-05-25

**Authors:** Ahmed Eid, Ayman Halabya, Nabil M. Nagy, Abbas Atef Hassan

**Affiliations:** 1 Department of Civil Engineering, Military Technical College, Cairo, Egypt; 2 Department of Architecture and Urban Planning, Port Said University, Port Said, Egypt

**Keywords:** Construction delays, Project management, Delay factors, Empirical validation, Contractor performance, Engineering, Mathematics and computing

## Abstract

Construction delays remain a major challenge, especially in developing countries where financial, administrative, and resource constraints intensify schedule disruptions. This study identifies and prioritizes construction delay factors through a three-phase research framework consisting of a literature review, an expert survey evaluation, and validation using real-life construction projects in Egypt. First, a comprehensive review of global literature led to the identification of 98 delay factors, which were classified into four main categories: owner-related factors (25 factors, 26%), contractor-related factors (34 factors, 35%), consultant/design-related factors (14 factors, 14%), and external factors (25 factors, 26%). Second, an expert survey was conducted to evaluate the previously identified delay factors. This phase employed a composite scoring approach that integrates both the frequency of occurrence and the impact of each factor to rank the most critical delay drivers. The findings indicate that owner-related factors constitute the most significant sources of delay, accounting for approximately 50% of the top 22 critical factors. Finally, the reliability of these findings was validated using the top 22 factors and a dataset of 141 real construction projects, showing strong alignment between the survey-based rankings and actual outcomes. Key factors, such as frequent change orders and design modifications, remained top-ranked, while some factors ranked higher or lower in practice, indicating minor variations in their relative importance under real project conditions. The study contributes a validated dataset of delay factors derived from both expert evaluation and real project evidence, providing a strong foundation for future predictive modeling applications using artificial intelligence and machine learning.

## Introduction

Construction is a significant driver of the global economy, contributing over $10 trillion annually and accounting for approximately 13% of the world’s total GDP according to the World Bank^1^. Large-scale construction projects frequently experience delays that significantly affect timelines, costs, and overall project success. According to the Project Management Institute (PMI), 9 out of 10 of construction projects worldwide face delays, typically extending project durations by 20–30% and resulting in significant cost overruns^2^. These delays cause substantial financial losses and impact not only economic performance but also the quality of infrastructure and the broader business environment. Given the scale of construction delays and their economic implications, understanding construction delay factors is critical for improving project delivery, supporting economic growth, and reducing financial risks for both public and private stakeholders.

Numerous studies have focused on understanding and analyzing construction delay factors on a global scale. Kaming et al.^3^ identified key factors such as poor planning, inadequate scheduling, and insufficient resources. Similarly, Aibinu and Jagboro^4^ emphasized labor shortages, inefficient procurement processes, and financial mismanagement, particularly in developing regions, while Chan et al.^5^ highlighted regulatory delays, including lengthy permitting processes and compliance challenges, as major contributors to extended project timelines. Recent studies have also emphasized the influence of contractual structures and responsibility allocation on project delays and overall^6^.

Beyond general delay factors, the relationship between project complexity and delays has also been examined extensively. Le-Hoai et al.^7^ demonstrated that large-scale, complex projects are especially prone to delays due to the high number of stakeholders and the challenges of resource management. Pinto and Slevin^8^ further argued that project failure, including delays, often results from inadequate control mechanisms and poor communication among project teams, stressing that effective communication is critical in reducing uncertainty and mitigating risks.

In addition to these global perspectives, researchers have identified both global and local factors contributing to construction delays. Shukri and Youssef^9^ highlighted that while global causes such as poor planning, labor shortages, and financial mismanagement are present, local factors such as political instability, frequent policy changes, and bureaucratic inefficiencies in permitting can significantly exacerbate schedule delays. These local challenges often result in approval delays, regulatory uncertainty, funding constraints, and procurement disruptions, particularly when combined with volatility in material markets.

Abdel-Mohsen et al.^10^ identified local regulatory challenges, including complex and frequently changing building codes, as major causes of construction delays. They pointed to inadequate coordination among governmental bodies, inconsistent enforcement of regulations, and inefficiencies in the inspection process as significant barriers to timely project completion. Additionally, they found that delays in payment processes, particularly in public sector projects, caused labor shortages and supply chain disruptions, further extending project timelines. Similarly, recent empirical research in developing-country contexts has identified financial constraints, weak feasibility studies, and poor inter-organizational communication as major contributors to construction delays, while also highlighting the potential of Building Information Modeling (BIM) and integrated project delivery approaches to mitigate such issues^11,12^.

More recently, research has shifted toward the use of advanced analytical approaches and data-driven techniques to improve decision-making in construction management. For instance, game-theory-based frameworks have been applied to model strategic interactions among project stakeholders, enabling more structured analysis of cost allocation, risk sharing, and coordination under uncertainty in construction projects^13^. In parallel, machine learning techniques have increasingly been applied in construction research to analyze large project datasets and support predictive analysis of project risks and operational performance^14,15^. These approaches enable more accurate predictive modeling, enhance project coordination, and support proactive identification of potential risks and conflicts in construction environments^16,17^.

In addition, optimization-based and analytical frameworks have been introduced to enhance decision-making in construction management by balancing cost, time, and quality considerations and improving quality management under^18,19^.

Despite the valuable contributions of previous research, there remains a notable gap in the comprehensive evaluation and prioritization of construction delay factors. Most studies have identified a limited number of relevant delay factors, with limited effort to assess their relative significance or validate their impact using real construction project data. This highlights a critical need for research that not only examines delay factors but also provides empirically validated prioritization to support more reliable decision-making in construction project management.

To address this gap, this study adopts a structured framework integrating a global literature review, expert-based evaluation, and validation using real construction project data. The novelty of this research lies in (1) the consolidation of 98 delay factors into a unified classification framework, (2) the development of a composite scoring approach integrating frequency and impact, and (3) the validation of the prioritized factors using data from 141 construction projects. The resulting validated dataset provides a reliable basis for understanding the relative importance of delay factors and may support future predictive modeling applications.

## Objectives

The main goal of this research is to address the previously identified gaps by evaluating construction delay factors through the integration of expert survey responses and real construction project data. The study follows a structured three-stage research framework designed to identify delay factors reported in previous studies, evaluate their relative importance based on expert perspectives, and verify their occurrence using real construction project data.

Based on this framework, the study pursues the following objectives:

1. Identification of delay factors: To identify a comprehensive set of construction delay factors through an extensive review of existing literature.
2. Evaluation and prioritization of delay factors: To evaluate and prioritize the identified delay factors through a structured survey conducted among industry professionals, capturing expert opinions regarding the frequency and impact of these factors.
3. Validation using real project data: To validate the survey findings through the analysis of data from ongoing or completed construction projects, enabling the practical verification of the most critical delay causes in real-world construction environments.

## Identification and consolidation of delay factors

This step focuses on compiling a comprehensive and representative list of potential delay causes by combining insights from the existing literature. This step follows a structured process involving (1) identification of delay factors from the literature, (2) consolidation of overlapping factors, and (3) classification into unified categories. The aim is to capture both widely recognized factors and those more specific to contemporary construction practices.

**Fig. 1:**
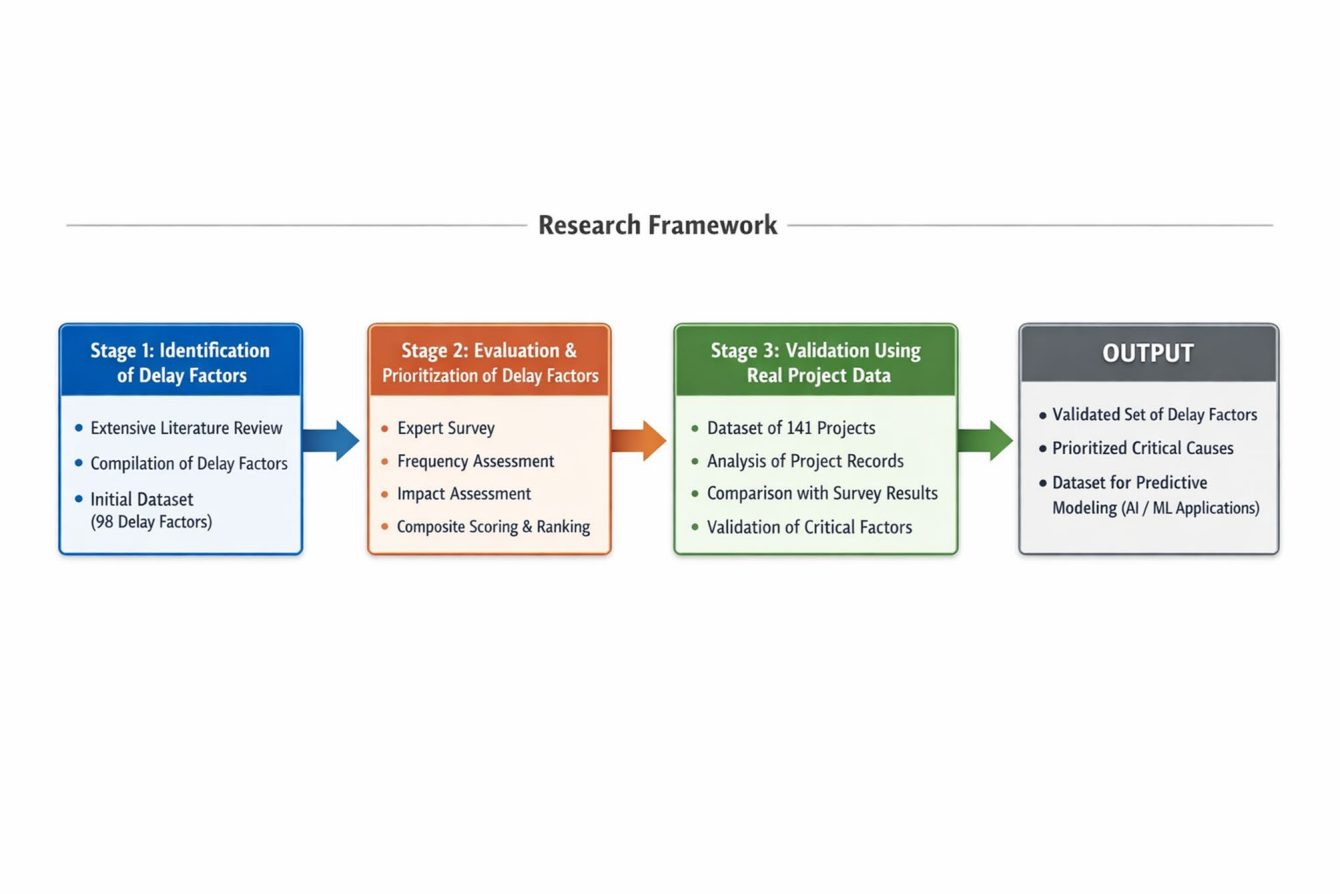
Research framework and methodology of the study.

To achieve this objective, 35 relevant studies were reviewed and analyzed, focusing on empirical studies, case analyses, and survey-based research from both global and regional contexts. The review covered sources that addressed general causes of construction delays, as well as those specific to developing and developed markets^20–27^. This process resulted in the extraction of 98 distinct delay factors from the literature. This process is illustrated within the overall research framework shown in Fig. 1, highlighting the transition from factor identification to classification and subsequent evaluation.

During the analysis, it became evident that previous studies have employed various approaches to classifying delay factors. Some studies categorized delays by the responsible party, such as owner-related, contractor-related, or consultant-related causes. Others adopted a legal or entitlement-based perspective, distinguishing between excusable and non-excusable delays and compensable and non-compensable delays. Additional classifications focused on project impact dimensions (e.g., cost, time, and quality) or project phases, including design, procurement, and construction.

To ensure consistency and comparability across studies, a unified classification approach was required. While existing categorization methods offer valuable insights, they often vary across studies and lack consistency. To create a unified framework suitable for further analysis and validation, this research consolidates the 98 identified delay factors into four main categories: (1) owner-related factors, representing delays attributed to the project owner’s role in the project lifecycle. These typically stem from decisions, actions, or administrative processes directly managed or controlled by the owner, (2) contractor-related factors, which include delays resulting from the contractor’s performance, planning, execution, or internal management throughout the construction phase, (3) design/consultant-related factors, referring to delays arising from the activities, coordination, or deliverables of designers, consultants, or engineering professionals involved in the technical development of the project, and (4) other project-related factors, which cover delays beyond the direct control of any single stakeholder. This final category also serves as a classification for any factors that do not clearly align with the owner, contractor, or consultant roles, including environmental, economic, and regulatory influences. This categorization enables a standardized comparison of delay factors across different studies and project contexts.

To assign each delay factor to the most appropriate category, a careful review was conducted based on the factor’s description and the stakeholder primarily responsible for its occurrence. This involved analyzing how each factor was presented in the original studies and determining which category it best aligned with. Because some delay factors may involve overlapping responsibilities among stakeholders (for example, between owners and consultants), classification was based on the primary source of occurrence within the project lifecycle. In such cases, the factor was assigned to the category representing the stakeholder from which the delay most commonly originates. In cases where a factor could reasonably fall under more than one category, comparisons across multiple sources were used to guide the classification. This methodological approach ensured that overlapping responsibilities were addressed consistently while maintaining a clear and practical classification structure.

Additionally, the factors within each category were ordered according to their frequency of appearance in the reviewed literature, with factors appearing more frequently placed closer to the top of the list.

This structured categorization offers a clear and comprehensive framework for organizing the identified delay factors, enabling systematic analysis, empirical validation, and the formulation of effective mitigation strategies. The full classification is presented in Table 4.

While this step provides a comprehensive and structured identification of delay factors, certain limitations should be acknowledged. The identification process is based on previously published studies, which may emphasize frequently reported factors while potentially overlooking emerging or project-specific causes. In addition, the consolidation and classification of delay factors rely on interpretation of the literature and the assignment of a primary responsible stakeholder, which may introduce a degree of subjectivity in cases of overlapping responsibilities. However, considerable effort was made to minimize these limitations by reviewing a wide range of studies from different contexts and by cross-checking factor classification across multiple sources to ensure consistency and reliability.

## Evaluation and prioritization of the identified construction delay factors

### Expert survey design and data collection

To evaluate and prioritize the previously identified delay factors, a structured process was followed. This involved collecting expert feedback through a targeted survey, calculating the frequency and impact of each factor, and identifying the most critical ones for further analysis. Before initiating the expert evaluation, a preliminary screening of the 98 delay factors was conducted to refine the list. This filtering step involved removing factors that were cited infrequently in the reviewed literature or were excluded due to their low likelihood of occurrence or limited applicability across typical project scenarios. This screening was conducted to ensure that the evaluation focused on delay factors that are commonly reported in construction projects and therefore more relevant for practical assessment by industry experts. As shown in Table 4, this review resulted in a refined list of 77 unique delay factors, which served as the basis for expert evaluation.

The evaluation process followed a structured workflow consisting of three main stages: (1) refinement of the initially identified delay factors through literature screening, (2) expert-based evaluation using a structured survey to assess the frequency and impact of each delay factor, and (3) quantitative prioritization through the calculation of normalized scores and composite scores. This step-by-step framework provides a systematic approach for identifying the most critical delay factors and ensures transparency in the evaluation and prioritization process.

To ensure the reliability of the results and broad representation of industry perspectives, participants were carefully selected to reflect a diverse range of professional roles and experience levels within the construction sector. The participant group included project managers, contractors, engineers, and consultants. This diversity was essential to support a more balanced and comprehensive evaluation of the identified delay factors. Prior to participation, all respondents were informed of the study objectives and methodology, and informed consent was obtained from each participant.

All survey responses were collected and analyzed in a de-identified and anonymized manner to ensure participant confidentiality. Participation was voluntary, and all personal information fields (e.g., name, company, project, mobile number, and email) were optional. The collected data were limited to general professional attributes (e.g., years of experience and role), and no sensitive personal data were obtained.

In accordance with the national research governance framework established by the Supreme Council of Universities, formal ethical approval is not required for studies of this nature that do not involve medical, clinical, or experimental procedures or the collection of sensitive personal data. The distribution of survey respondents by professional role and years of experience is presented in Tables 1 and 2, respectively.

**Table 1:** Experts’ professional roles distribution.

Experts’ profession	Number	Percentage
Owners	18	15.52
Managers	28	24.14
Consultants	24	20.69
Contractors	36	31.03
Designers	10	8.62
Total	116	100

**Table 2:** Profession of experts and distribution.

Years of experience	Number	Percentage
01 : 05 years	10	8.62
05 : 10 years	19	16.38
10 : 15 years	42	36.21
15 + years	45	38.79
Total	116	100

**Fig. 2:**
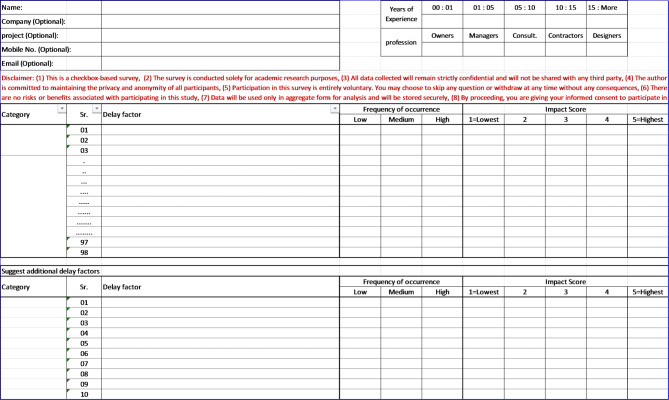
Survey template.

To collect expert input, a structured, paper-based checkbox-based survey was designed and administered in hard copy. The survey was printed on A3 sheets to provide a clear and spacious layout, enabling ease of completion. It featured a table listing all 77 delay factors in the first column, followed by three columns for rating the frequency of occurrence using a three-point Likert scale weighted as (1 = rarely, 3 = occasionally, 5 = frequently) and five columns for rating the impact using a five-point Likert scale (1 = lowest, 5 = very high). The weighting factors (1–3–5) were selected to provide clearer differentiation between the three levels of occurrence while maintaining a simple ordinal structure suitable for comparative analysis. This weighted scoring approach allows qualitative expert opinions to be converted into quantitative indicators for ranking delay factors and has been widely used in construction delay studies for evaluating the relative importance of project risks and delay causes. At the top of the form, a brief section was included to capture background information such as the participant’s name, company, project name, years of experience, and professional role, with stakeholder type (owner, contractor, consultant, or designer) as shown in Fig. 2.

The surveys were distributed and collected in person to ensure complete response and provide an opportunity to clarify any uncertainties during the process. This method helped maintain the quality of the data collected. In addition to the structured rating sections, the survey included open-ended fields allowing experts to suggest additional delay factors or provide contextual insights not captured in the predefined list. These dual inputs formed the basis for the prioritization process and set the stage for the subsequent phase of validation using real project data.

To assess the internal consistency and reliability of the survey responses, Cronbach’s alpha was calculated for the collected data. The resulting coefficient exceeded the commonly accepted threshold of 0.70, indicating satisfactory reliability of the survey instrument.

### Composite scoring and prioritization method

To systematically assess the significance of each identified delay factor, a composite scoring approach was employed, integrating both the frequency of occurrence and the impact score. This approach allows for a more comprehensive evaluation by considering not only how often a delay factor appears in projects but also the severity of its consequences on project timelines. By combining these two critical dimensions, the analysis provides a quantitative basis for ranking delay factors in terms of their overall risk to project performance.

The first step involved calculating individual scores for each delay factor based on its frequency of occurrence. A weighted scoring system was applied, where rarely occurring events were assigned a weight of 1, occasionally occurring events a weight of 3, and frequently occurring events a weight of 5. The frequency score (${F}_{i}$) for each factor was then determined by summing the weighted responses, as shown in Eq. (1). A similar procedure was followed to evaluate the impact of each delay factor. The impact score (${I}_{i}$) was calculated by summing the weighted values assigned to each impact level, as illustrated in Eq. (2), in order to standardize and compare the impact across all factors.

1 $${F}_{i}={n}_{1}\times 1+{n}_{2}\times 3+ {n}_{3}\times 5$$

where ${F}_{i}$ is the raw weighted frequency score for delay factor *i*, n1, n2, n3 represent the number of respondents who selected frequency ratings of 1 (rare occurrence), 2 (occasional occurrence), and 3 (frequent occurrence), respectively.

2 $${I}_{i}={m}_{1}\times 1+{m}_{2}\times 2+ {m}_{3}\times 3+ {m}_{4}\times 4+ {m}_{5}\times 5$$

where ${I}_{i}$ is the raw weighted impact score for delay factor *i*, m1, m2, m3, m4, m5 represent the number of respondents who selected impact ratings of 1 (lowest impact), 2 (low impact), 3 (average impact), 4 (high impact), and 5 (highest impact), respectively.

However, to ensure comparability across all delay factors, a min–max normalization was performed for the dataset, as shown in Eqs. (2), (3) and (4):

3 $${NF}_{i}= \frac{{F}_{i}-{Min}_{i=1}^{I}({F}_{i})}{{Max}_{i=1}^{I}({F}_{i})-{Min}_{i=1}^{I}({F}_{i})}$$

where ${NF}_{i}$ represents the normalized frequency score for delay factor *i*, ${Min}_{i=1}^{I}({F}_{i})$ is the minimum raw frequency score among (I) delay factors (I = 98 in our case), ${Max}_{i=1}^{I}({F}_{i})$ is the maximum frequency score among (I) delay factors (I = 98 in our case).

4 $${NI}_{i}= \frac{{I}_{i}-{Min}_{i=1}^{I}({I}_{i})}{{Max}_{i=1}^{I}({I}_{i})-{Min}_{i=1}^{I}({I}_{i})}$$

where ${NI}_{i}$ represents the normalized impact score for delay factor *i*, ${Min}_{i=1}^{I}({I}_{i})$ is the minimum raw impact score among (I) delay factors (I = 98 in our case), ${Max}_{i=1}^{I}({I}_{i})$ is the maximum impact score among (I) delay factors (I = 98 in our case).

This transformation scales both the frequency and impact scores between 0 and 1, where factors with the highest values are mapped closer to 1 and those with the lowest are mapped closer to 0. Following this normalization, a composite score (CS) was calculated to quantify the overall significance of each delay factor. The composite score was derived as the product of the raw normalized frequency score (${NF}_{i}$) and the normalized impact score (${NI}_{i}$) as shown in Eq. (5).

5 $${CS}_{i}={NF}_{i}\times {NI}_{i}$$

where ${CS}_{i}$ represents the Composite score, ${NI}_{i}$ is the normalized impact score*,* and ${NF}_{i}$ shows the normalized frequency score for delay factor *i.*

The composite score offers a balanced evaluation by integrating both the likelihood of occurrence and the severity of impact for each delay factor. Higher composite scores highlight factors that are not only frequent but also have serious consequences, making them priority issues for mitigation. The final ranking of delay factors was derived from these scores, allowing decision-makers to identify and focus on the most critical contributors to project delays. This methodology ensures a quantitative, structured, and objective assessment, applying min–max normalization to remove bias from extreme values while preserving the relative importance of each factor. As reflected in the resulting rankings Table 5, the evaluation supports an informed prioritization process and helps project managers design targeted strategies to reduce delays effectively. These prioritized factors form the basis for the subsequent validation phase using real construction project data.

### Results and discussion of prioritized delay factors

The analysis of CS rankings reveals several key trends that are consistent with findings reported in previous construction delay studies. Delay factors predominantly stem from Owner and Contractor sources. Among all evaluated factors, the most influential contributor was Frequent Change Orders and Design Modifications, which achieved a composite score of 100%, indicating both high frequency of occurrence and significant impact across surveyed projects.

Another critical owner-related factor was Delays in Progress Payments and Funding Issues (CS = 69.4%), underscoring the role of financial flow stability in project continuity. Similarly, Slow Decision-Making and Bureaucratic Processes (CS = 72.6%) and Delays in Approving Design Documents (CS = 62.5%) reflect inefficiencies in administrative and approval processes, which often result in idle time and hinder project momentum.

These findings are consistent with previous studies in construction management literature, which frequently identify owner-related factors, particularly change orders, delayed approvals, and decision-making processes, as dominant contributors to project delays [20], [27]. This consistency reinforces the reliability of the proposed prioritization approach and highlights the recurring nature of these delay drivers across different project contexts.

Contractor-side issues were also prominent. These included Delays in Manufacturing, Delivery, and Procurement of Materials (CS = 28.9%), Labor Shortages and Slow Mobilization (CS = 28.7%), and Inefficient Cost Control and Project Management (CS = 28.2%). These results indicate that, while owner-related factors dominate in terms of impact, contractor-related factors represent critical operational challenges that directly affect project execution efficiency.

From the Design/Consultants category, several factors emerged as moderately influential. Notably, Insufficient Data Collection and Survey Before Design (CS = 44.7%) and Inaccurate Site Investigations (CS = 39.5%) were ranked among the top third of all factors. These results highlight the importance of adequate due diligence and technical feasibility studies during early project phases. Other notable design-related factors included Poor Communication Between Owner and Contractor (CS = 30.7%) and Mistakes and Delays in Producing Design Documents (CS = 27.3%), suggesting a need for more integrated project delivery approaches and better information flow across stakeholders.

While external factors were generally less frequent, a few were found to have high impact when they did occur. Price Fluctuations (CS = 92.7%) and Unstable Exchange Rates and Material Costs (CS = 59.7%) indicate that macroeconomic volatility can significantly disrupt budgeting and procurement plans. Complexity of Project (CS = 34.0%) and Accidents During Construction (CS = 29.5%) also appeared among the higher-impact external risks.

Conversely, the lowest-ranked 20 delay factors, those with CS values below 2% were considered to have minimal influence on project timelines. These included items such as Conflicts in Joint Ownership and Decision-Making (CS = 0.6%), Weak or Ineffective Delay Penalty Mechanisms (CS = 0.6%), Personal Conflicts Among Labor (CS = 1.7%), and various rare external risks such as Pandemics and Force Majeure Events (CS = 1.2%), Sudden Failures from External Sources (CS = 0.0%), and Cultural or Religious Activity Conflicts (Not scored). Their low composite rankings reflect either their infrequent occurrence or limited perceived impact, supporting their deprioritization in strategic mitigation planning. These prioritized factors form the basis for the subsequent validation phase using real project data.

## Validation of prioritized delay factors using real project data

The third objective of this research is to validate the top delay factors previously identified and prioritized through expert evaluation by examining real-life data from selected construction projects. While expert opinion offers valuable insights into the perceived drivers of delay, validation using actual project documentation is essential to confirm the practical relevance and frequency of these factors across diverse project environments.

To achieve this, a targeted review was conducted using key project records, including Extension of Time (EOT) claims submitted by contractors, delay assessments issued by employers and project management consultants (PMCs), weekly and monthly progress reports, and lessons learned documentation. The analysis also considered supporting correspondence such as delay-related letters, formal notices of claim submitted by vendors, and instructions or clarifications issued by owners or PMCs. These documents provided traceable, firsthand evidence of delay causes, enabling a direct comparison between the prioritized factors and the delay events substantiated in practice. The use of multiple documentary sources allowed cross-verification of delay events and reduced the risk of relying on a single type of project record.

A refined list of 22 delay factors, representing those with the highest composite scores from the expert prioritization phase, was selected for validation, as shown in Table 5. These factors represent the most impactful and frequently cited contributors to construction delays. Focusing on this subset ensured that the analysis targeted only the most influential causes. Lower-ranking factors were excluded due to their limited frequency or reduced significance across real-world scenarios. This selection approach allowed the validation stage to concentrate on the delay factors most likely to influence project performance.

For this purpose, a dataset of 141 construction projects located in Egypt was compiled, encompassing a wide range of project types, including infrastructure, residential, and commercial developments. These projects were implemented across multiple regions within the country and represent typical construction activities in both urban and developing areas. The sample reflects diverse characteristics in terms of contract values, durations, project sizes, and contractual arrangements, and includes a range of project delivery models. The projects were executed by both local and international contractors, typically under the supervision of well-established engineering consultants. Most of the projects were commissioned by private-sector clients, while a smaller portion involved public or semi-governmental entities operating under the Egyptian construction regulatory framework.

The dataset was compiled from project records obtained through professional project management and consultancy practices, where the authors had access to project documentation as part of professional planning, project controls, and claims management activities. All project data were compiled and analyzed in an anonymized manner to ensure confidentiality of the organizations involved.

Each project record was reviewed in detail, capturing key attributes such as the project identification code and title, contractor and consultant names, and contractor tier classification, in addition to both planned and actual schedule data. Further information included observed schedule slippage, contract value changes, and project completion status, as well as the assignment of critical roles such as planners and contract administrators. The availability of supporting project controls and claims-related documentation, such as baseline schedules, schedule updates, periodic progress reports, extension of time (EOT) claims, and formal delay assessments, was also systematically recorded, as summarized in Table 3. These documents formed the primary basis for validating the occurrence of delay factors identified during the earlier stages of the study.

**Table 3:** Projects attributes and records.

Category item	Residential	Infra & roads	Comm	Grading	Total
No. of projects	75	39	7	20	141
% of overall	53.2%	27.7%	5.0%	14.2%	100%
No. of mega projects > 500M EGP	25	0	0	0	25
No. of large projects (Between 100 and 500M EGP)	28	7	0	8	43
No. of medium projects (Between 10 and 100 M)	14	22	2	12	50
No. of small projects (< 10 M EGP)	8	10	1	0	19
No. of projects in City Center	69	39	4	20	132
No. of projects in border	6	0	0	0	6
No. of projects with contractors Tier (1)	60	20	1	6	87
No. of projects with contractors Tier (2 & 3)	0	1	0	13	14
No. of projects with contractors Tier (4 & 5)	6	10	4	1	21
No. of projects with contractors Tier (6 & 7)	9	8	2	0	19
No. of projects with Planner/Controls Assigned	58	12	1	0	71
No. of projects with BL Schedule	75	29	7	3	114
No. of projects with Periodic Reports submitted	70	25	7	0	102
No. of projects with notices of claim/delay (correspondences)	1	0	0	0	1
No. of projects with EOT Claims	65	23	7	0	95
No. of projects with Other Claims	10	21	6	1	38
No. of projects with Delay Assessment reports	63	22	5	0	90

This rich and structured dataset provided the foundation for validating the selected delay factors. Each project was individually assessed using a set of predefined criteria. A factor was classified as “present” (marked as 1) if it was explicitly referenced in any of the project records noted above. It was marked as “absent” (marked as 0) if there was no documented evidence supporting its occurrence. This approach ensured consistency in evaluating the presence of delay factors across all projects.

**Fig. 3:**
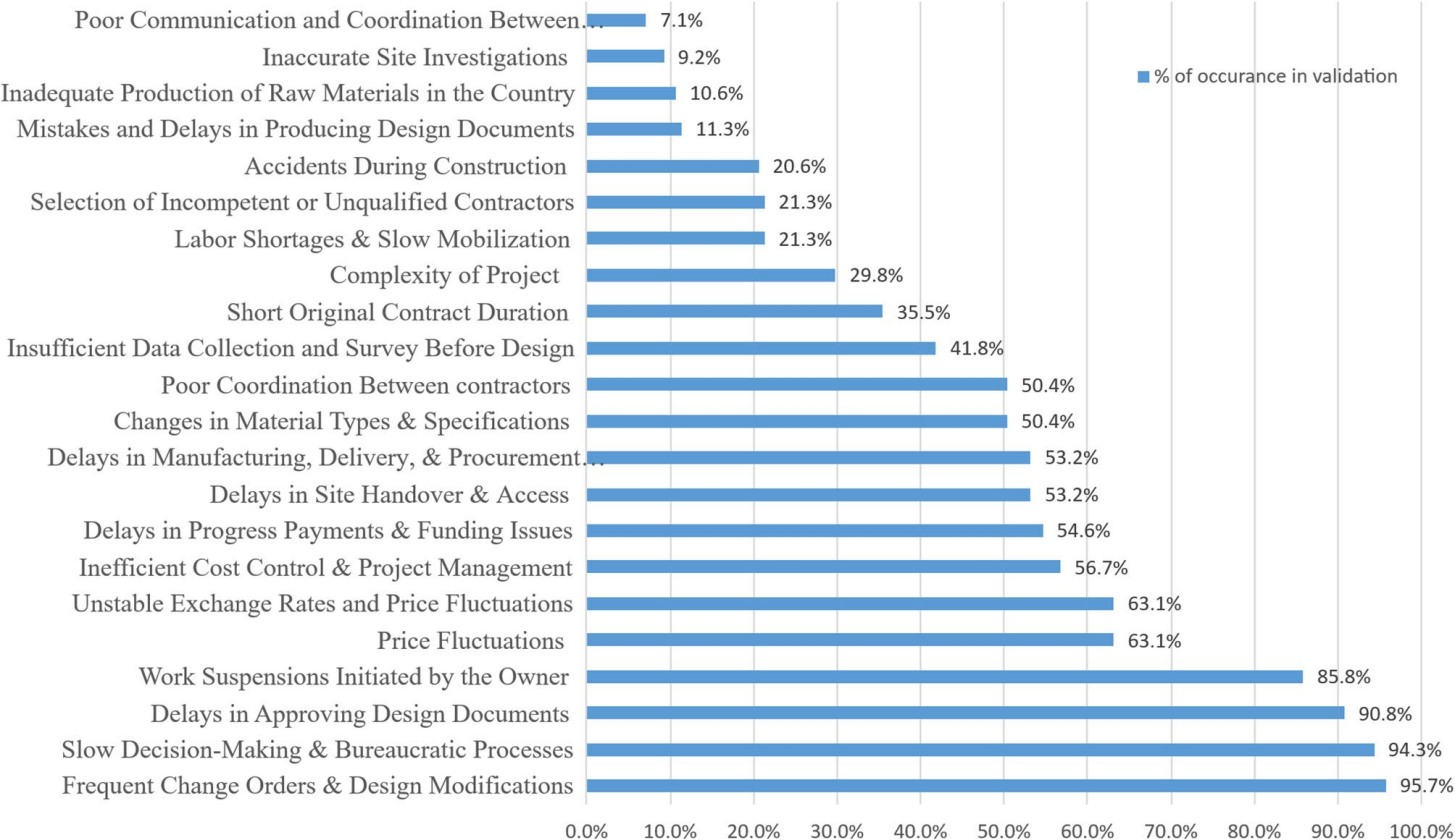
Percentage of recorded delay factors in assessed projects.

A validation matrix was developed to track the classification of each factor across all reviewed projects. The matrix structure allowed the aggregation of occurrences for each delay factor and facilitated the comparison between the expert-based prioritization results and the evidence obtained from real project data. The frequency of occurrence of each validated factor across the 141 projects was then calculated and compared with the rankings obtained from the expert survey to assess the level of agreement between expert perception and observed project data. This allowed trend analysis, revealing which delay factors consistently emerged in real project environments and which appeared less frequently than suggested by expert perception as shown in Fig. 3.

The matrix revealed a strong alignment in several key factors. “Frequent Change Orders & Design Modifications” was ranked first in both the survey-based and validation-based assessments, affirming its critical influence on construction delays. Similarly, “Delays in Approving Design Documents” and “Slow Decision-Making & Bureaucratic Processes” maintained top-tier positions, reflecting their consistent and significant impact across both expert opinion and empirical project data.

These findings are consistent with numerous previous studies in construction management literature, which identify change orders, delayed approvals, and owner decision-making processes among the most influential drivers of project delays. For example, studies by Abdulaziz Al-Momani [27] reported that design changes and slow client decisions frequently rank among the most critical delay causes in construction projects across different regions. Similar conclusions were also reported by Sadi A. Assaf and Saad Al-Hejji [20], who identified change orders and administrative delays as dominant contributors to schedule overruns in large construction projects. Comparable findings were also discussed by Samer Ezeldin (2013), who emphasized the significant impact of change orders and decision-making delays on construction project performance.

Despite this overall consistency, certain factors demonstrated noteworthy shifts in ranking. For instance, “Work Suspensions Initiated by the Owner” moved from a survey-based rank of 10th to 4th in the validation results. This suggests that the actual disruption caused by such suspensions may have been underestimated by experts. Likewise, “Inefficient Cost Control & Project Management” showed a substantial rise from 19th in the survey to 7th post-validation, indicating its more prominent role in project delays than initially perceived.

On the other hand, several factors experienced a drop in their relative importance when evaluated against real project data. “Delays in Progress Payments & Funding Issues”, initially ranked 4th by experts, fell to 8th in the validation ranking. A more pronounced shift was observed in “Poor Communication and Coordination Between Owner and Contractor”, which dropped from 15th to 22nd, indicating a lesser impact in practice than anticipated in the survey responses.

Overall, this comparison highlights a generally strong correlation between expert judgment and empirical evidence, particularly for the top-ranked factors. At the same time, it underscores the importance of validation using actual project records to uncover critical factors that may be underappreciated in perception-based assessments. The differences observed emphasize the dynamic nature of delay causation in construction and support the case for evidence-driven prioritization in risk mitigation strategies.

The analysis also showed that “frequent change orders and design modifications”, “slow decision-making and bureaucratic processes”, and “delays in approving design documents” were among the most consistently observed factors across projects of all sizes. These were particularly pronounced in large and medium-scale projects, where project complexity and stakeholder involvement are typically higher, leading to more frequent design iterations and administrative bottlenecks. For instance, large projects alone accounted for 45 instances of both change orders and slow decision-making, highlighting the management burden that often accompanies expansive scopes.

Interestingly, delays in progress payments and funding issues, though ranked fourth by experts, appeared more commonly in medium and small projects, while being almost absent in mega projects. This likely reflects the greater financial fragility or tighter cash flows experienced by smaller-scale developments, where contractors and owners are more susceptible to liquidity challenges.

When examining contractor classification, projects executed by lower-tier contractors (Tiers 4–7) tended to report higher occurrences of issues related to inefficient project management, labor shortages, and incompetent contractor selection. These findings align with expectations, as lower-tier firms often face resource limitations and may lack the robust systems and experience needed for effective execution. In contrast, Tier 1 contractors demonstrated fewer instances of such delays, though they were still affected by owner-driven delays such as late approvals and scope changes.

Project type also influenced delay patterns. Residential and infrastructure projects, which dominated the dataset, showed a higher prevalence of technical and administrative delays. For instance, delays in site handover and issues with design approvals were far more common in infrastructure projects, often due to land acquisition complications or regulatory interfaces. On the other hand, commercial projects, despite being fewer in number, showed a concentrated presence of planning and procurement-related delays, suggesting that tight timelines and fast-track expectations in commercial developments amplify certain risks.

A review of the documentation also indicated that projects with assigned planning and controls teams and proper baseline schedules were better equipped to formally record delay events, leading to more transparent claims and structured assessments. However, this also revealed a discrepancy: some factors that may exist in reality, such as labor shortages or external disruptions were underreported in documentation-heavy projects, possibly due to contractual sensitivities or reporting limitations.

To further interpret these findings and highlight their broader impact, the theoretical and practical implications are discussed. From a theoretical perspective, this study contributes to construction management research by providing a structured and validated framework for identifying, prioritizing, and verifying construction delay factors. The integration of literature-based identification, expert evaluation, and empirical validation enhances the reliability of delay assessment and supports the development of predictive and analytical models.

Additionally, from a practical perspective, the validated prioritization model supports decision-making by enabling stakeholders to focus on the most critical delay drivers, such as change orders, delayed approvals, and slow decision-making. It also assists project managers and planners in developing targeted mitigation strategies by aligning risk management efforts with the most impactful delay factors. For example, projects prone to financial or administrative delays can benefit from improved cash flow planning, stricter contract management, and clearer allocation of responsibilities, leading to more efficient resource use and improved project performance.

Furthermore, the validated dataset developed in this study provides a reliable foundation for future applications of machine learning and artificial intelligence in construction project management. The structured data, combining expert evaluation and real project validation, can support predictive models for delay forecasting and risk assessment and can be expanded in future research to improve accuracy and generalizability.

## Conclusion

To improve delay control in construction projects, this study identified and ranked 98 delay factors through a three-phase methodology consisting of literature-based identification, expert evaluation, and validation through real project data. The factors were grouped into four main categories: owner-related, contractor-related, consultant/design-related, and external factors.

Expert input from 116 professionals was analyzed using normalized frequency, impact, and composite scores, leading to a prioritized list of delay causes. Validation across 141 construction projects confirmed that owner-related issues, such as frequent change orders, slow decision-making, and delayed approvals, were the most influential. Contractor-side delays, planning inefficiencies, and consultant-related deficiencies also played significant roles, while certain external risks, although less frequent, had significant impacts when present.

The study highlights the importance of integrating expert insight with actual project documentation to accurately assess delay causes. From a theoretical perspective, the study reinforces the proposed framework by confirming its applicability through real project validation. From a practical perspective, the findings support improved prioritization and mitigation of delay factors in construction projects. The validated dataset also provides a basis for future AI- or ML-based delay prediction models.

While the study analyzed a relatively large dataset of construction projects covering different contractor tiers, project sizes, and project characteristics, certain limitations should be acknowledged. In particular, a higher proportion of the analyzed projects were residential developments, which may influence the relative representation of some delay factors. Nevertheless, the diversity of project characteristics included in the dataset supports the robustness of the findings and their relevance for broader construction project management practices.

## Data Availability

The datasets used and/or analyzed during the current study are available from the corresponding author upon reasonable request.

## Appendix A

See Tables 4 and 5.

**Table 4:** Identified delay factors classification.

Category	ID	Delay factor	Considered in evaluation
Owners	01	Unfavorable Contract Clauses	Yes
Owners	02	Short Original Contract Duration	Yes
Owners	03	Frequent Change Orders & Design Modifications	Yes
Owners	04	Conflicts in Joint Ownership & Decision-Making	Yes
Owners	05	Delays in Approving Design Documents	Yes
Owners	06	Delays in Progress Payments & Funding Issues	Yes
Owners	07	Delays in Site Handover & Access	Yes
Owners	08	Inaccurate or Inadequate Feasibility Studies	Yes
Owners	09	Lack of a Competent Owner’s Representative	Yes
Owners	10	Limited Owner Experience in Construction Projects	Yes
Owners	11	Lack of Incentives for Contractors to Expedite Work	Yes
Owners	12	Slow Decision-Making & Bureaucratic Processes	Yes
Owners	13	Work Suspensions Initiated by the Owner	Yes
Owners	14	Poor Planning & Project Management	Yes
Owners	15	Inefficient Financing & Payment Structures	Yes
Owners	16	Extended Time Between Design Completion & Tendering	Yes
Owners	17	Inappropriate Contractual Framework & Procedures	Yes
Owners	18	Selection of Incompetent or Unqualified Contractors	Yes
Owners	19	Failure to Obtain Necessary Permits on Time	Yes
Owners	20	Unclear Definition of Project Completion Criteria	Yes
Owners	21	Weak or Ineffective Delay Penalty Mechanisms	Yes
Owners	22	Changes in Material Types & Specifications	Yes
Owners	23	Poor Coordination Between contractors	Yes
Contractors	24	Frequent Subcontractor Changes	Yes
Contractors	25	Inadequate Contractor Experience	Yes
Contractors	26	Inefficient or Inappropriate Construction Methods	Yes
Contractors	27	Incompetent Project Team	Yes
Contractors	28	Ineffective Project Planning & Scheduling	Yes
Contractors	29	Obsolete Technology & Equipment	Yes
Contractors	30	Poor Communication and Coordination with Owner & Consultant	Yes
Contractors	31	Rework Due to Errors & Mistakes	Yes
Contractors	32	Unreliable Subcontractors & Suppliers	Yes
Contractors	33	Inadequate Site Investigations	Yes
Contractors	34	Inappropriate Contractor Policies & Procedures	Yes
Contractors	35	Poor Financial Control & Oversight on Site	Yes
Contractors	36	Poor Site Management & Supervision	Yes
Contractors	37	Equipment Allocation & Mobilization Issues	Yes
Contractors	38	Frequent Equipment Breakdowns & Low Efficiency	Yes
Contractors	39	Delays in Manufacturing, Delivery, & Procurement of Materials	Yes
Contractors	40	Material Shortages & Low-Quality Materials	Yes
Contractors	41	Labor Absenteeism & Low Productivity	Yes
Contractors	42	Personal Conflicts Among Labor	Yes
Contractors	43	Labor Shortages & Slow Mobilization	Yes
Contractors	44	Labor Strikes or Industrial Actions	Yes
Contractors	45	Unqualified or Inexperienced Labor	Yes
Contractors	46	Labor Injuries on Site	Yes
Contractors	47	Low Labor Motivation & Morale	Yes
Contractors	48	Slow Decision-Making & Delays in internal Approvals	Yes
Contractors	49	Inefficient Cost Control & Project Management	Yes
Design/Consultants	50	Lack of Consultant Experience in Construction Projects	Yes
Design/Consultants	51	Delay in Approving Major Changes in Scope of Work	Yes
Design/Consultants	52	Inaccurate Site Investigations	Yes
Design/Consultants	53	Inadequate Project Management Support	Yes
Design/Consultants	54	Poor Communication and Coordination Between Owner and Contractor	Yes
Design/Consultants	55	Conflicts Between Consultant and Design Engineer	Yes
Design/Consultants	56	Deficiencies in Team Experience and Procedures	Yes
Design/Consultants	57	Insufficient Data Collection and Survey Before Design	Yes
Design/Consultants	58	Mistakes and Delays in Producing Design Documents	Yes
Design/Consultants	59	Misunderstanding of Owner’s Requirements by Design Engineer	Yes
Design/Consultants	60	Poor Use of Advanced Engineering Design Software	Yes
External	61	Complexity of Project	Yes
External	62	Accidents During Construction	Yes
External	63	Delay in Performing Final Inspection and Certification by Third Party	Yes
External	64	Global Financial Crisis	Yes
External	65	Traffic Control and Restrictions at Job Site	Yes
External	66	Price Fluctuations	Yes
External	67	Neighbor-Related Issues	Yes
External	68	Unexpected Surface and Subsurface Conditions (e.g., Soil, Water Table)	Yes
External	69	Inadequate Production of Raw Materials in the Country	Yes
External	70	Thefts on Site	Yes
External	71	Changes in Government Regulations and Laws	Yes
External	72	Delay in Providing Services from Utilities (e.g., Water, Electricity)	Yes
External	73	Sudden Failures or Failures from External Sources	Yes
External	74	Unfavorable Weather Conditions	Yes
External	75	Inappropriate Government Policies	Yes
External	76	Unstable Exchange Rates and Price Fluctuations	Yes
External	77	Pandemics, Wars, and Other Force Majeure Events	Yes
Design/Consultants	78	Unclear and inadequate details in drawings	No
Contractors	79	Quality assurance/control	No
Design/Consultants	80	The slow reply to questionnaires by the contractor	No
Contractors	81	Delay in preparation of shop drawings and material samples	No
External	82	Environmental restrictions	No
Design/Consultants	83	Inflexibility of supervision	No
Contractors	84	Weakness of documented systems nin the consultant office	No
Contractors	85	Unreliable quantities takeoff	No
Contractors	86	Unreasonable site layout of prefabricated components or other materials	No
Contractors	87	Errors occurred during the installation, hoisting, and node connections of prefabricated components	No
Owners	88	Limited working space hindered hoisting and other construction activities	No
Contractors	89	Prefabricated components or materials were damaged or did not meet specifications	No
External	90	Cultural or Religious Activity Conflicts	No
owners	91	Errors in Legal Land Ownership or Boundary Disputes	No
External	92	Excessive Noise Complaints from Neighbors	No
External	93	Unavailability of Construction Water	No
External	94	Site Vandalism or Theft Leading to Major Delays	No
External	95	Shortage of Office Supplies or Admin Equipment	No
External	96	Unusual Wildlife Encounters on Site	No
External	97	Difference in culture and communication language	No
Contractors	98	Overly Detailed Site Safety Audits	No

**Table 5:** Overall composite score and ranking of time delay factors.

Category	ID	Rank	Delay factor	NF	NI	CS	Considered in Validation
Owners	03	01	Frequent Change Orders & Design Modifications	100.0%	100.0%	100.0%	Yes
External	66	02	Price Fluctuations	100.0%	92.7%	92.7%	Yes
Owners	12	03	Slow Decision-Making & Bureaucratic Processes	98.2%	74.0%	72.6%	Yes
Owners	06	04	Delays in Progress Payments & Funding Issues	69.4%	100.0%	69.4%	Yes
Owners	05	05	Delays in Approving Design Documents	100.0%	62.5%	62.5%	Yes
External	76	06	Unstable Exchange Rates and Price Fluctuations	91.0%	65.6%	59.7%	Yes
Owners	22	07	Changes in Material Types & Specifications	95.5%	53.1%	50.7%	Yes
Owners	07	08	Delays in Site Handover & Access	73.0%	64.6%	47.1%	Yes
Design / Consultants	57	09	Insufficient Data Collection and Survey Before Design	61.3%	72.9%	44.7%	Yes
Owners	13	10	Work Suspensions Initiated by the Owner	61.3%	71.9%	44.0%	Yes
Owners	23	11	Poor Coordination Between contractors	64.4%	65.6%	42.3%	Yes
Owners	02	12	Short Original Contract Duration	65.8%	63.5%	41.8%	Yes
Design / Consultants	52	13	Inaccurate Site Investigations	48.6%	81.3%	39.5%	Yes
External	61	14	Complexity of Project	45.9%	74.0%	34.0%	Yes
Design / Consultants	54	15	Poor Communication and Coordination Between Owner and Contractor	46.8%	65.6%	30.7%	Yes
External	62	16	Accidents During Construction	64.4%	45.8%	29.5%	Yes
Contractors	39	17	Delays in Manufacturing, Delivery, & Procurement of Materials	45.5%	63.5%	28.9%	Yes
Contractors	43	18	Labor Shortages & Slow Mobilization	45.9%	62.5%	28.7%	Yes
Contractors	49	19	Inefficient Cost Control & Project Management	31.5%	89.6%	28.2%	Yes
Owners	18	20	Selection of Incompetent or Unqualified Contractors	30.9%	89.6%	27.6%	Yes
Design / Consultants	58	21	Mistakes and Delays in Producing Design Documents	50.9%	53.6%	27.3%	Yes
External	69	22	Inadequate Production of Raw Materials in the Country	48.6%	55.2%	26.9%	Yes
Contractors	35	23	Poor Financial Control & Oversight on Site	42.8%	58.3%	25.0%	No
Design / Consultants	60	24	Poor Use of Advanced Engineering Design Software	45.0%	53.6%	24.2%	No
Contractors	28	25	Ineffective Project Planning & Scheduling	37.8%	63.5%	24.0%	No
Contractors	48	26	Slow Decision-Making & Delays in internal Approvals	31.5%	74.0%	23.3%	No
Design / Consultants	56	27	Deficiencies in Team Experience and Procedures	29.7%	75.0%	22.3%	No
Design / Consultants	55	28	Conflicts Between Consultant and Design Engineer	37.8%	56.3%	21.3%	No
Contractors	32	29	Unreliable Subcontractors & Suppliers	24.3%	75.0%	18.2%	No
Contractors	41	30	Labor Absenteeism & Low Productivity	31.5%	45.8%	14.5%	No
Owners	14	31	Poor Planning & Project Management	51.4%	28.1%	14.4%	No
External	68	32	Unexpected Surface and Subsurface Conditions (e.g., Soil, Water Table)	19.8%	66.7%	13.2%	No
Contractors	38	33	Frequent Equipment Breakdowns & Low Efficiency	16.2%	79.2%	12.8%	No
Contractors	33	34	Inadequate Site Investigations	18.9%	55.2%	10.4%	No
Contractors	37	35	Equipment Allocation & Mobilization Issues	13.5%	72.9%	9.9%	No
Contractors	31	36	Rework Due to Errors & Mistakes	9.9%	90.6%	9.0%	No
Contractors	36	37	Poor Site Management & Supervision	9.0%	95.3%	8.6%	No
Contractors	30	38	Poor Communication and Coordination with Owner & Consultant	13.5%	60.9%	8.2%	No
Contractors	25	39	Inadequate Contractor Experience	9.9%	82.3%	8.2%	No
Contractors	27	40	Incompetent Project Team	9.9%	82.3%	8.2%	No
External	64	41	Global Financial Crisis	9.9%	80.2%	7.9%	No
Contractors	40	42	Material Shortages & Low-Quality Materials	10.8%	72.9%	7.9%	No
Owners	19	43	Failure to Obtain Necessary Permits on Time	11.7%	67.2%	7.9%	No
Owners	01	44	Unfavorable Contract Clauses	14.4%	54.2%	7.8%	No
External	71	45	Changes in Government Regulations and Laws	15.3%	50.0%	7.7%	No
Design / Consultants	53	46	Inadequate Project Management Support	11.7%	53.6%	6.3%	No
Contractors	34	47	Inappropriate Contractor Policies & Procedures	9.9%	61.5%	6.1%	No
External	74	48	Unfavorable Weather Conditions	13.5%	39.6%	5.3%	No
Contractors	44	49	Labor Strikes or Industrial Actions	9.9%	51.6%	5.1%	No
Contractors	29	50	Obsolete Technology & Equipment	11.3%	43.8%	4.9%	No
Owners	20	51	Unclear Definition of Project Completion Criteria	8.1%	58.9%	4.8%	No
Design / Consultants	59	52	Misunderstanding of Owner’s Requirements by Design Engineer	6.3%	73.4%	4.6%	No
Design / Consultants	50	53	Lack of Consultant Experience in Construction Projects	6.3%	70.3%	4.4%	No
External	63	54	Delay in Performing Final Inspection and Certification by Third Party	6.3%	63.5%	4.0%	No
External	72	55	Delay in Providing Services from Utilities (e.g., Water, Electricity)	4.5%	69.8%	3.1%	No
Contractors	24	56	Frequent Subcontractor Changes	4.5%	67.7%	3.0%	No
External	70	57	Thefts on Site	9.9%	24.0%	2.4%	No
Owners	17	58	Inappropriate Contractual Framework & Procedures	5.9%	40.1%	2.3%	No
Owners	15	59	Inefficient Financing & Payment Structures	2.7%	74.0%	2.0%	No
External	75	60	Inappropriate Government Policies	3.6%	48.4%	1.7%	No
Owners	08	61	Inaccurate or Inadequate Feasibility Studies	2.7%	62.5%	1.7%	No
Contractors	42	62	Personal Conflicts Among Labor	6.8%	25.0%	1.7%	No
External	77	63	Pandemics, Wars, and Other Force Majeure Events	1.8%	65.6%	1.2%	No
Contractors	47	64	Low Labor Motivation & Morale	1.8%	43.8%	0.8%	No
Contractors	26	65	Inefficient or Inappropriate Construction Methods	0.9%	76.0%	0.7%	No
Contractors	45	66	Unqualified or Inexperienced Labor	0.9%	76.0%	0.7%	No
Owners	21	67	Weak or Ineffective Delay Penalty Mechanisms	0.9%	69.8%	0.6%	No
Owners	04	68	Conflicts in Joint Ownership & Decision-Making	6.3%	9.4%	0.6%	No
Owners	16	69	Extended Time Between Design Completion & Tendering	0.9%	55.2%	0.5%	No
Contractors	46	70	Labor Injuries on Site	1.8%	17.7%	0.3%	No
Owners	09	71	Lack of a Competent Owner’s Representative	4.5%	0.0%	0.0%	No
Owners	10	72	Limited Owner Experience in Construction Projects	16.2%	0.0%	0.0%	No
Owners	11	73	Lack of Incentives for Contractors to Expedite Work	2.7%	0.0%	0.0%	No
Design / Consultants	51	74	Delay in Approving Major Changes in Scope of Work	0.0%	75.5%	0.0%	No
External	65	75	Traffic Control and Restrictions at Job Site	14.4%	0.0%	0.0%	No
External	67	76	Neighbor-Related Issues	6.3%	0.0%	0.0%	No
External	73	77	Sudden Failures or Failures from External Sources	0.0%	89.1%	0.0%	No
